# Professional identity and sense of coherence affect the between compassion fatigue and work engagement among Chinese hospital nurses

**DOI:** 10.1186/s12912-023-01596-z

**Published:** 2023-12-13

**Authors:** Yiming Zhang, Qianwen Peng, Wanglin Dong, Cui Hou, Chaoran Chen

**Affiliations:** 1 Institute of Nursing and Health, School of Nursing and Health, Henan University, Kaifeng, People’s Republic of China; 2 Department of Health and Wellness, Nanyang Vocational College of Science and Technology, Nanyang, People’s Republic of China

**Keywords:** Compassion fatigue, Professional identity, Sense of coherence, Work engagement, Clinical nurses

## Abstract

**Background:**

With the continuous improvement of people’s health needs, the public’s requirements for medical care are also getting higher and higher. Work engagement is a positive psychological state related to the work. It is very important to maintain nurses’ work engagement, however, due to many factors, the level of nurses’ work engagement is not high and nursing managers should identify the influencing factors of work engagement, and take positive measures to fully improve nurses’ work engagement.

**Objectives:**

To explore the influence of compassion fatigue, professional identity and sense of coherence on nurses’ work engagement.

**Methods:**

From January 2022 to June 2022, convenience sampling was used to select clinical nurses from 9 tertiary hospitals in Henan Province of China as the research objects for a questionnaire survey. Statistical methods included descriptive statistical analysis, Pearson correlation analysis and the PROCESS Macro Model 4 and 7 in regression analysis.

**Results:**

The results showed that compassion fatigue was significantly negatively correlated with sense of coherence, professional identity and work engagement (*P*<0.01), professional identity was significantly positively correlated with sense of coherence and work engagement (*P*<0.01), and there was a significant positive correlation between sense of coherence and work engagement (*P*<0.01). Professional identity played a partial mediating role between compassion fatigue and work engagement, accounting for 46.40% of the total effect; meanwhile, sense of coherence moderated the effect of compassion fatigue on professional identity and formed a moderated mediation model.

**Conclusions:**

Compassion fatigue has a negative predictive effect on nurses’ work engagement. Professional identity and sense of coherence further explained the relationship of compassion fatigue on compassion fatigue and work engagement through mediating and moderating effects.

**Supplementary Information:**

The online version contains supplementary material available at 10.1186/s12912-023-01596-z.

## Introduction

Clinical nurses are an important occupational group in medical and health institutions and play an important role in providing medical and health services [1]. However, most countries are facing a shortage of nurses. The World Health Organization predicts that the global shortage of nurses will reach 5.7 million by 2030 [2]. In addition, due to the particularity, high risk and high load of nursing work, many nurses are under high work pressure and workload, and are prone to job burnout and turnover intention [3], which leads to a more serious shortage of nursing human resources. The shortage of nursing human resources aggravated the work burden of nurses, which made many nurses unable to fully devote themselves to work. Work engagement is most often defined as ‘’a positive, fulfilling, work-related state of mind characterized by vigour, dedication, and absorption’’ [4]. Previous studies have shown that work engagement is a positive psychological state related to work, which can not only effectively reduce nurses’ job burnout and turnover intention, but also further improve nurses’ satisfaction with nursing work and improve their job performance and nursing service quality [5, 6]. Understanding the status of nurses’ work engagement and exploring the factors affecting nurses’ work engagement are not only the focus of current nursing managers, but also very important for improving the quality of nursing service and promoting the healthy development of nursing.

Researchers have conducted extensive exploration on the factors that may affect nurses’ work engagement, and found that nurses’ work engagement is not only affected by external factors such as organizational climate, leadership style, workload [1, 5, 7], but also closely related to individual subjective psychological factors [8]. As providers of health care, nurses aim to provide care, support, and assistance to patients according to their physical, psychological, emotional, and spiritual needs. However, nurses have to face the pain of patients every day in the process of caring for patients, and have compassion for the pain suffered by patients. In the long run, nurses will have job burnout due to excessive compassion investment, and their compassion for the pain suffered by patients will be reduced, and compassion fatigue will occur [9]. Compassion fatigue is defined as the negative emotional consequences of chronic and repeated exposure to the mental and physical suffering of others in the provision of care [10]. As an important stress response, compassion fatigue has received considerable attention among medical workers [11]. In the study of Korean nurses, compassion fatigue has a significant positive predictive effect on work stress and is a significant negative predictor of job satisfaction [12]. A study of Australian nurses showed that compassion fatigue was significantly and positively correlated with high levels of anxiety and depression [13]. In the study of physicians and nurses in China, it was found that compassion fatigue was closely related to burnout and depression of physicians and nurses [14]. Compassion fatigue of nurses is closely related to the level of work engagement [15]. However, there are few studies on the overall relationship between compassion fatigue and work engagement, and the process of the relationship between the two is still unclear, which needs further in-depth research.

Professional identity is defined as an individual’s understanding of their own profession in professional practice, and it is a feeling and cognition of the professional value of their profession and the development of their personal ability in the profession [16]. According to the three-dimensional professional identity theory [17], an individual’s recognition of professional value and role value will be enhanced through the development of individual cognition, emotion and intentional behavior, thereby improving individual work engagement. Relevant studies have shown that nurses’ compassion fatigue can affect nurses’ professional identity [18], and professional identity plays a crucial role in nurses’ work engagement [19]. Therefore, nurses’ professional identity may be an important mediating variable in the relationship between compassion fatigue and work engagement. Furthermore, nurses’ own internal psychological resources may moderate this process. Sense of coherence is defined as a person’s general orientation towards life that guides people to find and use resources to stay healthy, especially during times of considerable strain, which helps to cope with life stress [20]. It refers to the ability of individuals to maintain their physical and mental health in the face of stressful situations in work and daily life. It is not only an important psychological resource for medical and health personnel, but also an important predictor of nurses’ professional identity [21, 22]. Thus, as a positive psychological resource, nurses’ sense of coherence may play a moderating role in this process.

According to the job demand resource theory, nurses need to compassion to the patients they are caring for in the nursing work. In the long run, nurses’ energy can be eroded by their compassion for patients, leading to problems such as poor job performance and work engagement. Professional identity and sense of coherence, as important psychological factors of individuals, are also regarded as the work resources of nurses in nursing work, and may play an important role in the relationship between compassion fatigue and work engagement of nurses. Existing studies have examined the relationship between compassion fatigue and work engagement, the relationship between compassion fatigue and professional identity, and the relationship between professional identity and work engagement [15, 18, 23]. But through a search of the relevant literature, we found that there is no study on the relationship between the four variables of compassion fatigue, professional identity, sense of coherence and work engagement, especially in clinical nurses. Based on this, this study used the independent variable of compassion fatigue, professional identity as the mediating variable, sense of coherence as the moderating variable, and work engagement as the dependent variable to construct and verify the mediating effect model, in order to provide new ideas and suggestions for hospitals and nursing managers to further improve the work engagement of clinical nurses, and provide reference and empirical basis for improving the level of nursing service.

## Theoretical basis

**Fig. 1:**
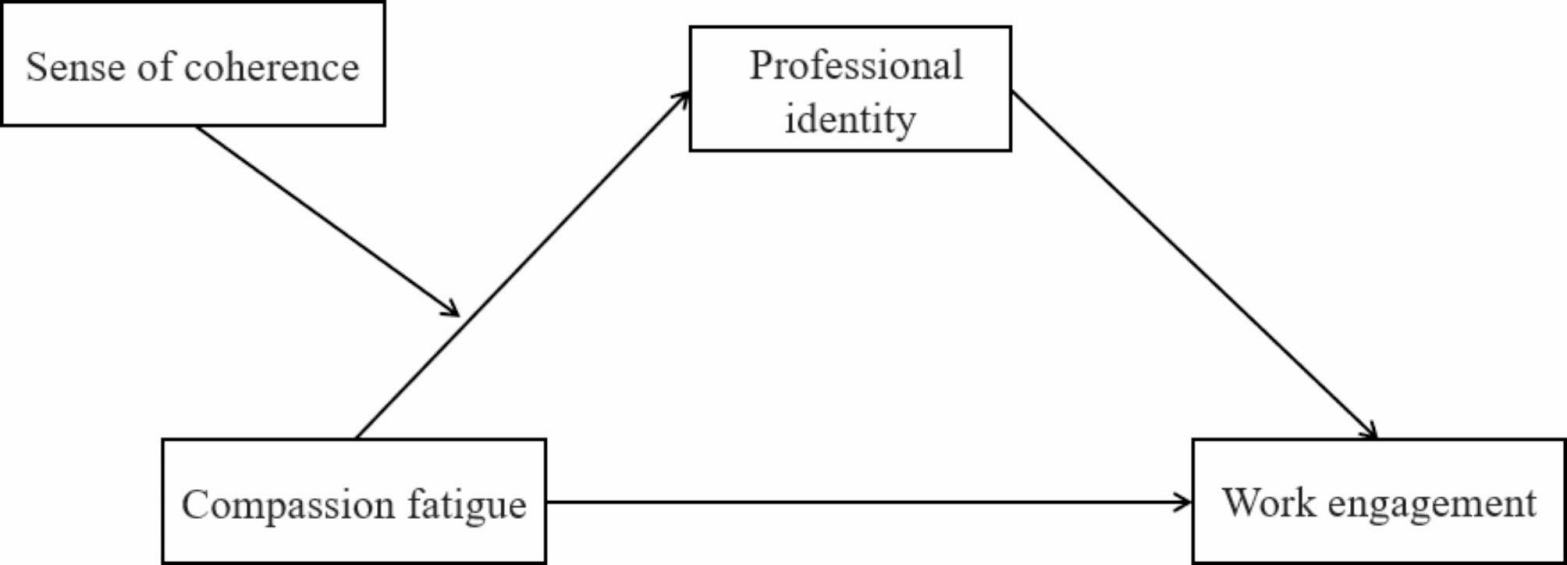
The theoretical model of this study

The job demand-resource theory is proposed by Demerouti [24]. This model believes that the job characteristics of each occupation will be expressed in two forms: job requirements and job resources. Among them, job demand refers to the individual’s physical, cognitive or emotional investment in the process of work due to the job demand. Work resources refer to those resources that have an incentive effect on individual growth and can promote individuals to achieve work goals. Job demands and job resources can affect job burnout and job engagement through two different pathways, namely “fatigue process” and “motivation process”. The “fatigue process” refers to the process by which an employee’s original energy is eroded by constant effort to cope with long-term job demands, leading to poor performance, decreased job engagement, and physical and mental health problems. “Motivational process” refers to the process of improving the degree of work engagement, work enthusiasm and work performance of individuals due to the sufficiency of work resources. Compassion plays the role of “job demand” in the job demand-resource model. Therefore, when nurses pay too much compassion and appear compassion fatigue, it will lead to the decline of individual work engagement. Professional identity acts as a “job resource”, which can promote individuals to cope with job requirements, complete work tasks, and improve individual work engagement. The sense of coherence can make individuals identify, benefit from, and control and use their own resources, so it may play a moderate role in individual resources. Based on the above theoretical basis, this study designed the research hypothesis model as shown in Fig. 1, and put forward the following hypotheses:

H1. Compassion fatigue has a direct negative impact on nurses’ work engagement.

H2. Compassion fatigue has a direct negative impact on nurses’ professional identity.

H3. Professional identity has a direct positive impact on nurses’ work engagement.

H4. Professional identity plays a mediating role between compassion fatigue and work.

engagement.

H5. Sense of coherence plays a moderating role between professional identity and work engagement.

## Methods

### Design and participants

Using the convenient sampling method, a questionnaire was distributed to clinical nurses who met the inclusion criteria from 9 tertiary hospitals in Henan Province from January 2022 to June 2022. The nine surveyed tertiary hospitals were all general hospitals, mainly from three prefecture-level cities in Henan province, including four affiliated university hospitals. The number of hospital beds in the survey ranged from 1200 to 3541, and the annual outpatient visits ranged from 0.30 million to 1.90 million. The number of nurses participating in the survey in these 9 hospitals were 121, 182, 116, 158, 189, 207, 145, 138, 142, respectively. The inclusion criteria included (1) clinical registered nurses; (2) engaged in clinical nursing work for more than 1 year; (3) nurses with informed consent and voluntary cooperation to participate in this investigation. Nurses who were not directly involved in patient care and intern nurses were excluded. According to the sample size calculation formula *N* = 4Uα^2^S^2^/δ^2^ [25] and the results of the pre-survey, the standard deviation S was 1.63, and the allowable error δ was set as 0.2, α = 0.05, *N* = 4 × 1.96^2^ × 1.63^2^/0.2^2^≈1021. Due to the possibility of loss of follow-up and invalid questionnaires in the process of issuing questionnaires, 1398 questionnaires were actually issued after adding 20% of the sample size. 38 regular response questionnaires and 43 lost of follow-up were excluded, and 1317 valid questionnaires were finally collected, with an effective recovery rate of 94%.

### Data collection

On the basis of the agreement of the hospital nursing department and departments, the questionnaires were distributed to the nurses who met the inclusion and exclusion criteria. The purpose and precautions of this study were explained before the questionnaire was distributed, and the questionnaire could not be distributed until the informed consent of the nurses was obtained. The validity of the questionnaire was checked by two people on the spot when the questionnaire was collected to ensure the accuracy and completeness of the data.

### Measures

#### Demographic information questionnaire

Based on the research content and purpose of this study, the demographic data questionnaire of clinical nurses was designed by the researchers. There were 12 items in this part, which mainly investigated the age, gender, work departments, working years, professional title, education level, average weekly working hours, average monthly night shifts, average monthly income, marital status, number of children and mode of employment.

#### Compassion fatigue scale

The Chinese version of Compassion Fatigue Short Scale (C-CF) compiled by Adams [9] and revised by Lou [26]. The scale consisted of two dimensions of secondary trauma and job burnout, with a total of 13 items. Among them, secondary trauma was composed of 5 items, and job burnout was composed of 8 items. The scale was scored by Likert 10 points, and the full score was 130 points. The higher the total score, the more serious the degree of compassion fatigue. The level of compassion fatigue can be divided into three levels according to the average score of items: mild (< 4 points), moderate (4–7 points) and severe (> 7 points), and the questionnaire has good construct validity. The Cronbach’s α coefficient of the total scale in this study was 0.918.

#### Professional identity scale

The professional identity rating scale for nurses compiled by Liu [27] was adopted. The scale had 5 dimensions, a total of 30 items. The scale was scored by Likert 5-point scale, with a total score of 150 points, and the higher the score, the higher the professional identity. The Cronbach’s α coefficient of the scale in this study was 0.960.

#### Sense of coherence scale

Sense of coherence scale (SOC-13) with 13 items compiled by Antonovsky [28] was selected for measurement, and the Chinese version was revised by domestic scholar Bao [29]. In this scale, there are five reverse scoring items, and the remaining eight items are all positive scoring. The scale included 3 sub-dimensions of comprehensibility, manageability and meaning-fulness. Likert 7-point scale was used, and the full score was 91. A higher total score indicates a higher level of sense of coherence. The scale has good reliability and validity, and the criterion-related validity is ideal. The Cronbach’s α coefficient of the scale in this study was 0.870.

#### Work engagement scale

The short version of the 9-item Work Engagement Scale (UWES-9) compiled by Schaufeli [4] was used. The scale included 3 dimensions, and each dimension included 3 items. The scale uses a 7-point Likert scoring method, ranging from 0 (never) to 6 (every day), with a full score of 54. Higher scores indicate higher individual work engagement. According to the average score of the items, they could be divided into three levels: mild (< 2 points), moderate (2–4 points) and severe (> 4 points). The scale has good reliability and validity, and some domestic scholars have confirmed that the Chinese version of the scale has good reliability and validity [30]. The total Cronbach’s α coefficient of the scale in this study was 0.945.

### Ethical considerations

This study was reviewed and approved by the Sub-Committee of Biological Science Research Ethics of Henan University (No. HUSOM2021-286) and followed the ethical principles of informed consent, timely explanation was given to the investigators before the investigation, and informed consent was obtained before the investigation. Besides, this study was an anonymous survey, and all investigators could voluntarily withdraw from this study, and the data collected were strictly confidential and used only for this study.

### Data analysis

The statistical analyses were carried out using the SPSS 26.0 software and the PROCESS Macro. Frequency, mean and standard deviation were used for descriptive analysis, and the relationships among compassion fatigue, professional identity, sense of coherence and work engagement were analyzed by Pearson’s correlation analysis.PROCESS is a plug-in developed by Hayes that specializes in analyzing mediating and moderating effects [31]. According to the methods provided by Hayes [31], PROCESS Models 4 (to test mediating effects) and 7 (to test the moderating variable’s moderating effect on the first half path) in the SPSS regression analysis PROCESS v4.1 program were used to test the moderated mediation effect. Besides, in order to better explain the moderating effect of sense of coherence and determine whether the mediating effect varied with the moderating variable, we used the simple slope method and the 5000 resample bootstrapping method to divide the sense of coherence into high and low groups based on the mean +/ -1 SD of the sense of coherence [32, 33], and tested whether the mediating effect was significant in different level of the moderating variable. All statistical analyses within this study were two-sided, with a significance level of 0.05, and a P value of less than 0.05 was considered to indicate statistical significance.

## Results

### Participant characteristics

A total of 1317 valid questionnaires were collected in this survey, of which the proportion of female nurses was 80.4%. In terms of age, 39.0% of the nurses were between 26 and 35 years old. Most of the nurses worked in internal medicine and surgery departments, accounting for 28.0% and 25.7%, respectively. 40.5% of the nurses had worked for 1–5 years. The nurses with average monthly income of 6001–9000 yuan were the most, accounting for 34.1%. The results of participant characteristics are shown in Table 1.

**Table 1:** Descriptions of nurse characteristics (N = 1317) Continued Table 1

Socio-demographic characteristics		*N*	%
Age(years)	≤ 25	472	35.8
	26–35	513	39.0
	36–45	234	17.8
	>45	98	7.4
Gender	Male	158	19.6
	Female	1059	80.4
Education level	Junior college and below	492	38.7
	Bachelor degree	730	54.7
	Master degree or above	95	6.6
Professional title	Nurse	489	37.1
	Nurse practitioner	518	39.3
	Nurse-in-charge or above	310	23.5
Departments	Internal medicine	369	28
	Surgery	339	25.7
	Obstetrics and gynaecology	136	10.3
	Paediatrics	104	7.9
	Emergency	130	9.9
	Intensive care unit	121	9.2
	Others	118	9
Work experience (years)	1–5	534	40.5
	6–10	476	36.1
	11–15	186	14.1
	>15	121	9.2
Average working hours per week (hours)	≤ 40	295	22.4
	41–50	638	48.4
	>50	384	29.2
Average number of night shifts per month (times)	0–2	141	10.7
	3–5	340	25.8
	6–8	555	42.1
	>8	281	21.3
Average monthly income(¥)	≤ 3000	289	21.9
	3001–6000	326	24.8
	6001–9000	449	34.1
	>9000	253	19.2
Marital status	Single	526	39.9
	Married	707	53.7
	Divorced or widowed	84	6.4
Number of children	0	578	43.9
	1	531	40.3
	≥ 2	208	15.8
Mode of employment	Aurhorized personnel	238	18.1
	Personnel agency	338	25.7
	Contract worker	741	56.3

### Pearson’s correlation analysis

Means, standard deviations (SD), and Pearson correlations of each variable are shown in Table 2., among clinical nurses, the overall average scores of compassion fatigue, professional identity, sense of coherence and work engagement were (3.68 ± 1.84), (3.40 ± 0.59), (4.39 ± 0.92) and (3.57 ± 1.21) respectively. Pearson’ correlation analysis showed that the correlation coefficients of compassion fatigue, professional identity, sense of coherence and work engagement were − 0.565, 0.647, 0.431, respectively (P < 0.01). The correlation coefficient between professional identity and sense of coherence was 0.368 (P < 0.01), and the correlation coefficient between professional identity and compassion fatigue was − 0.555 (P < 0.01), and the correlation coefficient between sense of coherence and compassion fatigue was − 0.273 (P < 0.01).

**Table 2:** Descriptive statistics and correlation analysis (r)

Variables	$\bar x \pm s$	CF	PI	SOC	WE
CF	3.68 ± 1.84	1			
PI	3.40 ± 0.59	-0.555^**^	1		
SOC	4.39 ± 0.92	-0.273^**^	0.368^**^	1	
WE	3.57 ± 1.21	-0.565^**^	0.647^**^	0.431^**^	1

Abbreviations: CF, compassion fatigue; PI, professional identity; SOC, sense of coherence; WE, work engagement. ^**^P < 0.01

### Mediating effect with moderating analysis

**Fig. 2:**
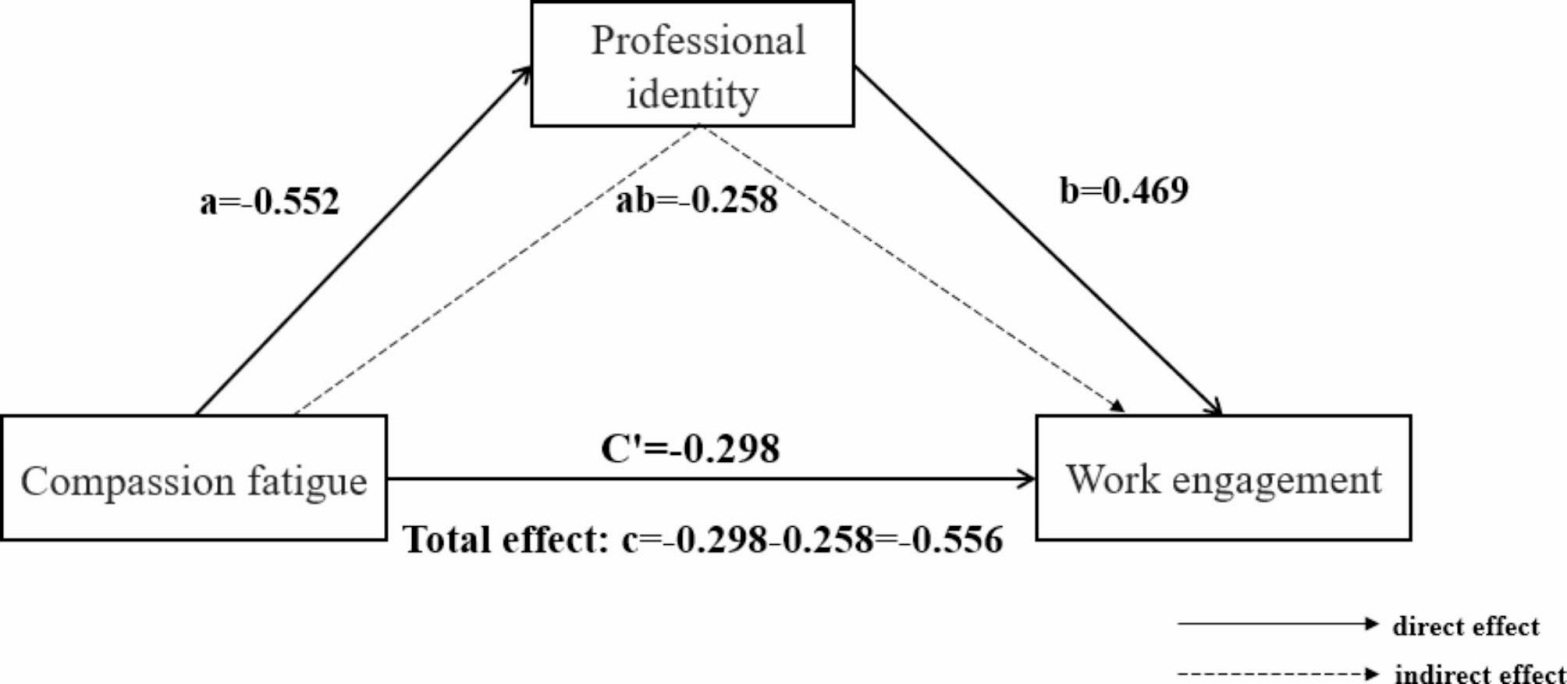
Mediating effect of professional identity

Through multiple linear regression analysis, it was found that professional title and average monthly income had a significant impact on work engagement, so they were used as control variables in the mediating effect. After the variables were standardized, PROCESS model 4 in SPSS was used to analyze the mediating effect, and the Bootstrap sampling number was set to 5000 times to test the mediating effect [31]. The results showed that after controlling for nurses’ title and average monthly income, compassion fatigue negatively predicted work engagement (c=-0.556, t=-24.823, P < 0.001). When compassion fatigue and professional identity were included in the regression equation, the effect size of compassion fatigue negatively predicting work engagement was reduced (c’=-0.299, t=-12.7589, P < 0.001), and compassion fatigue negatively predicted professional identity (a=-0.552, t=-24.121, P < 0.001). Professional identity also had a significant positive predictive effect on work engagement (b = 0.469, t = 19.727, P < 0.001). The Bootstrap test showed that professional identity had a significant mediating effect between compassion fatigue and work engagement (ab=-0.258, P < 0.001), Boot SE = 0.018, 95%CI [-0.296, -0.225], the proportion of mediating effect in the total effect was ab/ (ab + c’) = 46.40%, that is, in the relationship between compassion fatigue and work engagement of clinical nurses, professional identity played a part of the mediating effect, and the mediating effect accounted for 46.40% of the total effect. As shown in Fig. 2.

The PROCESS model 7 was used to analyze the moderating effect. The results showed that model 1 was significant (F = 202.272, P < 0.001, R^2^ = 0.316) when demographic variables were controlled. Compassion fatigue could negatively predict professional identity (β=-0.552, t=-24.121, P < 0.001), model 2 was significant (F = 319.427, P < 0.001, R^2^ = 0.493). Compassion fatigue could negatively predict work engagement (β=-0.298, t=-12.589, P < 0.001), and professional identity could positively predict work engagement (β = 0.469, t = 19.727, P < 0.001), model 3 was significant (F = 172.187, P < 0.001, R^2^ = 0.396). Compassion fatigue had a significant negative predictive effect on professional identity (β=-0.536, t=-23.340, P < 0.001), and the interaction term between compassion fatigue and sense of coherence was significant (β=-0.163, t=-8.427, P < 0.001), indicating that sense of coherence had a moderating effect on the relationship between compassion fatigue and professional identity in the above mediation model, as detailed in Table 3.

**Table 3:** The model of mediating effect with moderating

Predictive variable	Model 1(PI)				Model 2(WE)						Model 3(PI)		
	*β*	*SE*	*t*		*β*	*SE*	*t*			*β*		*SE*	*t*
Professional title	0.074	0.030	3.216^**^		0.093	0.026	3.607^***^			0.064		0.028	2.283^*^
Average monthly income	0.040	0.022	1.759		0.082	0.019	4.299^***^			0.027		0.021	1.310
CF	-0.552	0.023	-24.121^***^		-0.298	0.024	-12.589^***^			-0.536		0.023	-23.340^***^
PI					0.469	0.024	19.727^***^						
SOC										0.237		0.022	10.565^***^
CF*SOC										-0.163		0.019	-8.427^***^
*R* ^*2*^	0.316				0.493					0.396			
*F*	202.272^***^				319.427^***^					172.187^***^			

Abbreviations: CF, compassion fatigue; PI, professional identity; SOC, sense of coherence; WE, work engagement. ^*^P<0.05, ^**^P < 0.01, ^***^P < 0.001

**Fig. 3:**
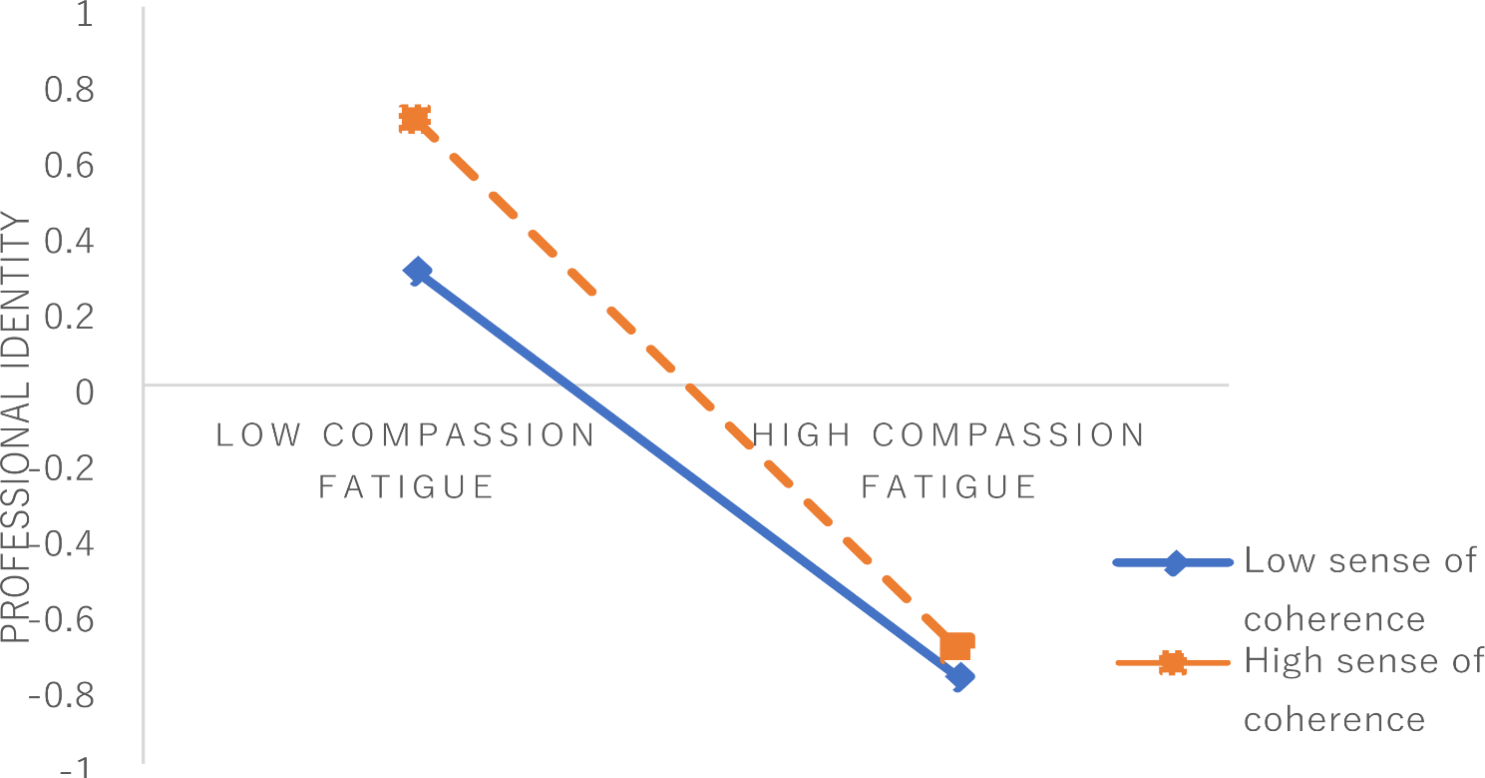
Moderating effect of sense of coherence

The level of sense of coherence was divided into high score group and low score group, and the simple effect slope figure as shown in Fig. 3. Clinical nurses with low score group (M-1SD), the impact of compassion fatigue on professional identity was negatively significant (β=-0.373, t=-14.192, P < 0.001). For clinical nurses with high level of sense of coherence (M + 1SD), compared clinical nurses who with the low level of sense of coherence, the negative slope of compassion fatigue on professional identity was greater (β=-0.699, t=-20.963, P < 0.001), see Table 4 for details. This means that under the same level of compassion fatigue, nurses with high sense of coherence can obtain more professional identity growth than nurses with low sense of coherence.

**Table 4:** Simple slope analysis

Sense of coherence	β	*SE*	t	*P*	*LLCI*	*ULCI*
M-1SD	-0.373	0.026	-14.192	<0.001	-0.425	-0.322
M	-0.536	0.023	-23.340	<0.001	-0.581	-0.491
M + 1SD	-0.699	0.033	-20.963	<0.001	-0.765	-0.634

## Discussion

This study explored the relationships among compassion fatigue, professional identity, sense of coherence and work engagement of nurses. The findings are consistent with the proposed theoretical framework. First, the result showed that compassion fatigue was negatively correlated with professional identity, sense of coherence and work engagement, while the remaining variables were positively correlated with each other. Second, the mediating effect showed that professional identity played a partial mediating effect between compassion fatigue and work engagement of clinical nurses. Third, sense of coherence has a significant moderating effect between compassion fatigue and professional identity, and under the same level of compassion fatigue, nurses with high level of sense of coherence can obtain more professional identity growth than those with low sense of coherence.

In this study, the compassion fatigue score was consistent with the study by Yang and Zhu [34], but higher than that in the study by Barnett [35] and Arıkan [36]. This difference may be related to the different nursing departments. In different departments, the severity of patients’ illness and the workload of nurses are different, which may cause the differences in compassion fatigue of nurses in different departments. The professional identity of clinical nurses is at a medium level, which is consistent with the research results of Yu [37]. It shows that nurses have certain cognition of nursing profession, but they still need to further improve their professional identity. In addition, the level of sense of coherence of clinical nurses in this study (57.06 ± 11.94) was lower than the score of nurses in developed countries Sweden (61.43 ± 0.76) and Spain (67.9 ± 10.02) [21, 38]. China has a large population, the aging population and the “two-child” policy have greatly increased the demand for nurses [39, 40]. Due to the shortage of nursing staff, Chinese nurses face heavier work and bear greater pressure than developed country. Previous studies have shown that sense of coherence is negatively correlated with job stress [41, 42]. These may be the reasons why the level of sense of coherence of nurses in China is lower than that in Sweden and Spain. Finally, nurses in this study had a moderate level of work engagement, which was lower than the studies of Baghdadi [43]and Borges [44]. The reason for the inconsistent results may be related to the hospital level. This study investigated a tertiary hospital in China. Compared with other levels of hospitals, tertiary hospitals have a large number of patients, more serious conditions, and a large nursing workload, which may also be the reason for lower results than in other studies.

The findings support H1 that compassion fatigue is a significant negative predictor of work engagement, which is consistent with the findings of Cao and Chen [15]. Compassion fatigue is a work-related stressor and easily lead to the consumption of individual psychological resources. According the conservation of resources theory [45], when nurses perceive compassion fatigue, they may need more resources to cope with compassion fatigue, and in order to further reduce the loss of their own resources, nurses are less engaged in their work, which results in lower work engagement [46]. In addition, according to the job demand-resource model [24], on the one hand, nurses need to have compassion for patients in the process of patient care, and compassion is a job requirement of nurses in nursing work. On the other hand, nurses need to be exposed to the trauma caused by patients’ pain for a long time in clinical nursing work, and are in the risk environment of secondary trauma. If they cannot be adjusted and recovered in time, they will have a negative impact on their physical and mental health [47], reduce their job satisfaction [48], produce job burnout [49], and treat their work with a negative attitude [14]. As a result, the level of work engagement of nurses decreased.

The results of this study show that compassion fatigue is significantly negatively correlated with professional identity, which supports H2 and is also consistent with previous research [50]. The study of Yi [18] showed that compassion fatigue of nursing interns reduced their enthusiasm for nursing and professional identity. The formation of professional identity is a continuous development process, and an individual’s educational experience, life experience, work experience and social media will all have an impact on their professional identity [18]. Relevant studies also believe that nurses’ basic values and the formation, development and maintenance of values will also have resulting in a profound impact on their practice [51, 52]. Geoffrion [51] believe that caregivers’ cognition of their profession will change their basic beliefs about the world due to long-term trauma, which in turn may endanger nurses’ self-cognition of the nursing profession, affect individuals’ attitudes, values and cognition of their profession, and have a negative impact on mental health. Therefore, nurses with higher compassion fatigue also had lower identification with their profession.

The positive correlation between professional identity and work engagement supports H3, indicating that the higher the level of professional identity of clinical nurses, the higher the level of work engagement, which is consistent with Zhang [23]. When individuals have a positive identity with their profession, they will devote more energy and enthusiasm to their work, and the dissatisfaction caused by the working environment will be eliminated to a certain extent [17]. According to self-determination theory [52], professional identity can affect internal cognition and enhance internal motivation through individual self-regulation, which has a positive role in promoting nurses’ work engagement. Therefore, with the continuous increase of nurses’ professional identity, the perceived work pressure and job burnout level of nurses in clinical nursing work also decreases, and their self-worth can also be reflected in the work. They often show positive attitudes and behaviors, and can put more enthusiasm and energy into clinical nursing work from the heart [53, 54]. Therefore, nurses with higher professional identity have stronger work enthusiasm and higher work engagement.

The mediating effect showed that professional identity played a partial mediating role in the relationship between compassion fatigue and professional identity of clinical nurses, and this result supported H4. The reason may be that the more serious the compassion fatigue of clinical nurses is, the weaker the perception and compassion of clinical patients are, and they cannot meet the emotional needs of patients in a timely and effective manner [11]. In addition, the long-term high-load, high-pressure and high-risk clinical work and the long-term exposure to patients’ pain and trauma environment, the original energy of nurses is eroded in order to cope with the work requirements [55]. With the continuous loss of their own resources, negative emotional problems gradually occurred, and their recognition of the nursing profession also decreased [56]. As a means of maintaining resources, professional identity can reduce the consumption of own resources. If the professional identity of clinical nurses decreases, the nurses’ sense of professional benefits will also decrease, and the fatigue and burnout will increase at work, and they will often adopt a negative attitude to deal with work, which will lead to the decrease of their work efficiency and work engagement [19]. On the contrary, when nurses’ professional identity is high, their dissatisfaction at work will be correspondingly reduced, and their perceived professional benefits in nursing work will be correspondingly increased, so as to reduce the negative impact of compassion fatigue on work engagement.

This study also supports H5 that sense of coherence played a moderating role between compassion fatigue and professional identity, and nurses with a high level of sense of coherence had a greater impact on professional identity than nurses with a low level of sense of coherence. This indicates that nurses with a high sense of coherence can obtain more career identity growth than those with a low sense of coherence at the same level of compassion fatigue. The reason may be that nurses with a high sense of coherence have a high level of professional identity, under the same level of compassion fatigue, nurses with high sense of coherence can get relief faster and better than those with low sense of coherence. Therefore, with the compassion fatigue decrease, the slope value of high sense of coherence in the simple slope adjustment effect chart is also larger. Nurses with a high sense of coherence can correctly evaluate stress, think that the stimulation and stress they suffer are predictable and interpretable, adopt more adaptive strategies to cope with the emerging compassion fatigue by regulating stressful events, and effectively extract and mobilize existing resources to cope with compassion fatigue [4, 19, 57]. Since nurses with a high sense of coherence have a high level of professional identity, slight changes in the face of compassion fatigue may cause large fluctuations in their professional identity level. Nurses with low sense of coherence have a low level of professional identity. Even with the alleviation of compassion fatigue, the change of professional identity is smaller than that of nurses with high sense of coherence. It’s important to note, through the simple moderating effect chart, it can be seen that no matter how the level of sense of coherence changes and how the slope value decreases, the level of professional identity of nurses with high sense of coherence is higher than that of nurses with low sense of coherence.

### Implication for nursing management

This study provides a new perspective for improving the level of nurses’ work engagement, and also provides a theoretical and practical basis for subsequent scholars to carry out further in-depth research on nurses’ work engagement. First, nurses can relieve compassion fatigue at work by communicating more with family and colleagues, participate in more positive psychological lectures, and continuously improve their nursing knowledge and skills to increase their coping ability and ability to deal with problems. Second, nursing managers should timely identify the tendency of compassion fatigue in nurses, and relieve nurses’ negative psychology and emotions through reasonable scheduling lecture on psychology and psychological counseling, so as to reduce nurses’ compassion fatigue. In addition, nursing managers should pay attention to the mediating role of professional identity and the moderating role of sense of coherence, build a good humanistic atmosphere of the department, provide a supportive working environment for clinical nurses, improve nurses’ working attitude by reasonable allocation of work resources (such as arrange shift patterns scientifically and allocate workforce rationally, and reduce nurses’ workload [58]), change the leadership style according to the characteristics and actual situation of nurses (such as studies have shown that transformational leadership can improve nurses’ work engagement [59]), strengthen training and education and other measures (such as implementation of programs to increase sense of coherence among nurses [60], support for staff education contributes to professional identity and practice [61]), so as to improve nurses’ work engagement. Finally, medical institutions should pay enough attention to nurses, provide training and promotion opportunities for nurses, meet the needs of nurses for career development, let nurses feel their working status has been improved, their self-worth has been affirmed, and reasonable allocation of nursing human resources according to workload and actual work needs, and further improve the welfare and work performance of nurses. Medical colleges should also pay attention to the cultivation of professional quality of nursing students, strengthen the education of professional value and sense of coherence.

### Limitations

This study has several limitations. First, the study was a cross-sectional study and was only investigated in a single time period. In future research, longitudinal studies can be conducted to understand and compare the dynamic development and changes of compassion fatigue, professional identity and work engagement in clinical nurses. ent. Second, according to hospital scale, research direction, human resources and technical strength, Chinese hospitals were divided into three levels, namely primary hospitals, secondary hospitals, and tertiary hospitals. The number of patients and the severity of the patient’s condition are different in primary, secondary and tertiary hospitals, the workload and work stress of nurses are also different. This study used convenience sampling and only investigated tertiary hospitals; primary and secondary hospitals were not involved. Different hospital characteristics, different levels of hospitals and convenient sampling methods may affect the measured variables in this study, resulting in underrepresentation of the sample and limiting the conclusions. Therefore, future studies should improve their design, adopt a multicenter design and include both primary and secondary hospitals to increase the representativeness and generalization of the results. Third, this study only investigated the relationship between compassion fatigue, sense of coherence, professional identity and work engagement, and has not explored the effects of other variables on nurses’ work engagement. The results of conceptual analysis and other study results suggest that personal-professional integrity, coping strategies resilience and self-efficacy are important influencing variable of work engagement [15, 62, 63]. Besides, resilience as a stress coping resource, it can effectively help buffer the negative impact of workplace stressors on nurses, and it closely intertwined with self-coherence, personal-professional integrity, compassion fatigue, and various other similar concepts within diverse nursing environments. Therefore, Future research should consider including more variables, especially resilience, to comprehensively explore the important factors affecting clinical nurses’ work engagement.

## Conclusion

Clinical nurses have a low level of compassion fatigue, and their professional identity, sense of coherence and work engagement are at a medium level. Compassion fatigue of clinical nurses was significantly correlated with professional identity, sense of coherence and work engagement. Professional identity plays a partial mediating role between compassion fatigue and work engagement in nurses, and sense of coherence plays a moderating role in the indirect path of compassion fatigue affecting nurses’ professional identity. Nursing managers should pay more attention to compassion fatigue of clinical nurses and pay attention to improving nurses’ professional identity and sense of coherence, so as to further improve their work engagement.

### Electronic supplementary material

Below is the link to the electronic supplementary material.

Supplementary Material 1

## Data Availability

The datasets can be made available to any interested person(s) contacting the corresponding author via email.
