# Supplementary material for: Professional identity and sense of coherence affect the between compassion fatigue and work engagement among Chinese hospital nurses
Source: BMC Nurs. 2023 Dec 13;22:472. doi: 10.1186/s12912-023-01596-z (PMC10717496; doi:10.1186/s12912-023-01596-z)
Supplement: Supplementary file 1 — Supplementary Material 1 [file 12912_2023_1596_MOESM1_ESM.docx]

**Table S1 Univariate analyses of compassion fatigue (N = 1317)**

| Variables | | *N* | Mean ± SD | *t*/*F* | *P* |
| --- | --- | --- | --- | --- | --- |
| Age(years) | ≤25 | 472 | 47.93±24.77 | 0.245 | 0.865 |
|  | 26-35 | 513 | 48.19±23.73 |  |  |
|  | 36-45 | 234 | 47.28±23.58 |  |  |
|  | ＞45 | 98 | 46.13±21.04 |  |  |
| Gender | Male | 158 | 49.69±21.54 | 1.429 | 0.153 |
|  | Female | 1059 | 47.32±24.40 |  |  |
| Education level | Junior college and below | 492 | 48.51±26.67 | 0.606 | 0.545 |
|  | Bachelor degree | 730 | 47.55±22.06 |  |  |
|  | Master degree or above | 95 | 45.75±22.05 |  |  |
| Professional title | Nurse | 489 | 48.18±24.45 | 0.156 | 0.856 |
|  | Nurse practitioner | 518 | 47.75±24.89 |  |  |
|  | Nurse-in-charge or above | 310 | 47.21±21.14 |  |  |
| Departments | Internal medicine | 369 | 47.09±22.81 | 0.724 | 0.630 |
|  | Surgery | 339 | 46.61±22.88 |  |  |
|  | Obstetrics and gynaecology | 136 | 48.79±28.36 |  |  |
|  | Paediatrics | 104 | 47.54±23.97 |  |  |
|  | Emergency | 130 | 49.78±26.35 |  |  |
|  | Intensive care unit | 121 | 50.81±23.17 |  |  |
|  | Others | 118 | 47.07±22.21 |  |  |
| Work experience (years) | 1-5 | 534 | 48.75±25.73 | 0.980 | 0.401 |
|  | 6-10 | 476 | 47.89±23.11 |  |  |
|  | 11-15 | 186 | 46.44±21.88 |  |  |
|  | ＞15 | 121 | 45.16±21.11 |  |  |
| Average working hours per week (hours) | ≤40 | 295 | 44.59±22.46 | 6.092 | 0.002 |
|  | 41-50 | 638 | 47.37±22.29 |  |  |
|  | ＞50 | 384 | 50.92±26.97 |  |  |
| Average number of night shifts per month (times) | 0-2 | 141 | 44.90±20.43 | 3.022 | 0.029 |
|  | 3-5 | 340 | 46.20±25.70 |  |  |
|  | 6-8 | 555 | 47.80±22.49 |  |  |
|  | ＞8 | 281 | 51.11±25.54 |  |  |

Continued Table S1

| Average monthly income（¥） | ≤3000 | 289 | 50.28±25.09 | 1.849 | 0.136 |
| --- | --- | --- | --- | --- | --- |
|  | 3001-6000 | 326 | 48.50±21.98 |  |  |
|  | 6001-9000 | 449 | 46.57±24.29 |  |  |
|  | ＞9000 | 253 | 46.18±24.06 |  |  |
| Marital status | Single | 526 | 48.18±25.47 | 0.500 | 0.606 |
|  | Married | 707 | 47.77±22.69 |  |  |
|  | Divorced or widowed | 84 | 45.37±23.47 |  |  |
| Number of children | 0 | 578 | 47.88±24.97 | 0.679 | 0.507 |
|  | 1 | 531 | 47.07±23.00 |  |  |
|  | ≥2 | 208 | 49.33±22.99 |  |  |
| Mode of employment | Aurhorized personnel | 238 | 45.87±21.63 | 1.480 | 0228 |
|  | Personnel agency | 338 | 47.07±24.05 |  |  |
|  | Contract worker | 741 | 48.72±24.46 |  |  |

**Table S2 Univariate analyses of professional identity (N = 1317)**

| Variables | | *N* | Mean ± SD) | *t*/*F* | *P* |
| --- | --- | --- | --- | --- | --- |
| Age(years) | ≤25 | 472 | 100.02±17.87 | 4.622 | 0.003 |
|  | 26-35 | 513 | 102.36±16.09 |  |  |
|  | 36-45 | 234 | 104.09±20.51 |  |  |
|  | ＞45 | 98 | 105.85±18.50 |  |  |
| Gender | Male | 158 | 99.25±17.56 | -2.861 | 0.004 |
|  | Female | 1059 | 102.78±17.84 |  |  |
| Education level | Junior college and below | 492 | 101.79±16.81 | 0.189 | 0.828 |
|  | Bachelor degree | 730 | 102.18±18.23 |  |  |
|  | Master degree or above | 95 | 102.95±19.93 |  |  |
| Professional title | Nurse | 489 | 100.00±16.61 | 5.571 | 0.004 |
|  | Nurse practitioner | 518 | 103.03±17.75 |  |  |
|  | Nurse-in-charge or above | 310 | 103.82±19.49 |  |  |
| Departments | Internal medicine | 369 | 101.86±17.42 | 2.053 | 0.056 |
|  | Surgery | 339 | 104.22±16.86 |  |  |
|  | Obstetrics and gynaecology | 136 | 101.92±21.10 |  |  |
|  | Paediatrics | 104 | 101.13±17.91 |  |  |
|  | Emergency | 130 | 98.89±18.18 |  |  |
|  | Intensive care unit | 121 | 99.79±18.09 |  |  |
|  | Others | 118 | 103.59±16.51 |  |  |
| Work experience (years) | 1-5 | 534 | 100.12±17.46 | 4.502 | 0.004 |
|  | 6-10 | 476 | 102.71±16.14 |  |  |
|  | 11-15 | 186 | 104.03±21.14 |  |  |
|  | ＞15 | 121 | 105.36±19.44 |  |  |
| Average working hours per week (hours) | ≤40 | 295 | 104.37±19.21 | 4.610 | 0.010 |
|  | 41-50 | 638 | 102.17±17.89 |  |  |
|  | ＞50 | 384 | 100.20±16.41 |  |  |
| Average number of night shifts per month (times) | 0-2 | 141 | 105.40±19.31 | 6.195 | ＜0.001 |
|  | 3-5 | 340 | 103.66±19.07 |  |  |
|  | 6-8 | 555 | 102.06±16.70 |  |  |
|  | ＞8 | 281 | 98.58±17.18 |  |  |

Continued Table S2

| Average monthly income（¥） | ≤3000 | 289 | 99.34±16.47 | 3.515 | 0.015 |
| --- | --- | --- | --- | --- | --- |
|  | 3001-6000 | 326 | 101.80±16.62 |  |  |
|  | 6001-9000 | 449 | 103.31±19.21 |  |  |
|  | ＞9000 | 253 | 103.43±18.03 |  |  |
| Marital status | Single | 526 | 101.74±16.65 | 0.284 | 0.753 |
|  | Married | 707 | 102.22±18.53 |  |  |
|  | Divorced or widowed | 84 | 103.20±19.16 |  |  |
| Number of children | 0 | 578 | 101.82±16.59 | 1.146 | 0.318 |
|  | 1 | 531 | 102.23±18.59 |  |  |
|  | ≥2 | 208 | 102.55±18.87 |  |  |
| Mode of employment | Aurhorized personnel | 238 | 105.05±20.57 | 5.703 | 0.003 |
|  | Personnel agency | 338 | 102.90±17.35 |  |  |
|  | Contract worker | 741 | 100.77±16.97 |  |  |

**Table S3 Univariate analyses of** **sense of coherence (N = 1317)**

| Variables | | *N* | Mean ± SD | *t*/*F* | *P* |
| --- | --- | --- | --- | --- | --- |
| Age(years) | ≤25 | 472 | 56.21±11.96 | 3.929 | 0.008 |
|  | 26-35 | 513 | 56.71±12.01 |  |  |
|  | 36-45 | 234 | 58.31±11.49 |  |  |
|  | ＞45 | 98 | 60.09±12.06 |  |  |
| Gender | Male | 158 | 58.75±10.78 | 2.731 | 0.007 |
|  | Female | 1059 | 56.65±12.18 |  |  |
| Education level | Junior college and below | 492 | 56.43±11.56 | 1.442 | 0.237 |
|  | Bachelor degree | 730 | 57.32±12.14 |  |  |
|  | Master degree or above | 95 | 58.38±12.34 |  |  |
| Professional title | Nurse | 489 | 55.73±11.82 | 4.933 | 0.007 |
|  | Nurse practitioner | 518 | 57.72±11.85 |  |  |
|  | Nurse-in-charge or above | 310 | 58.06±12.14 |  |  |
| Departments | Internal medicine | 369 | 57.04±11.84 | 0.848 | 0.533 |
|  | Surgery | 339 | 56.26±12.44 |  |  |
|  | Obstetrics and gynaecology | 136 | 57.03±12.51 |  |  |
|  | Paediatrics | 104 | 57.66±11.43 |  |  |
|  | Emergency | 130 | 56.88±11.68 |  |  |
|  | Intensive care unit | 121 | 57.21±11.83 |  |  |
|  | Others | 118 | 59.04±10.98 |  |  |
| Work experience (years) | 1-5 | 534 | 56.24±11.84 | 1.939 | 0.121 |
|  | 6-10 | 476 | 57.23±12.09 |  |  |
|  | 11-15 | 186 | 58.05±10.69 |  |  |
|  | ＞15 | 121 | 58.55±13.44 |  |  |
| Average working hours per week (hours) | ≤40 | 295 | 58.26±11.64 | 7.363 | 0.001 |
|  | 41-50 | 638 | 57.67±11.81 |  |  |
|  | ＞50 | 384 | 55.14±12.20 |  |  |
| Average number of night shifts per month (times) | 0-2 | 141 | 58.89±12.53 | 1.440 | 0.229 |
|  | 3-5 | 340 | 57.07±11.64 |  |  |
|  | 6-8 | 555 | 56.95±11.98 |  |  |
|  | ＞8 | 281 | 56.37±11.91 |  |  |

Continued Table S3

| Average monthly income（¥） | ≤3000 | 289 | 55.84±11.73 | 2.601 | 0.051 |
| --- | --- | --- | --- | --- | --- |
|  | 3001-6000 | 326 | 56.37±12.89 |  |  |
|  | 6001-9000 | 449 | 57.74±11.56 |  |  |
|  | ＞9000 | 253 | 58.17±11.47 |  |  |
| Marital status | Single | 526 | 56.21±11.77 | 2.437 | 0.088 |
|  | Married | 707 | 57.55±12.02 |  |  |
|  | Divorced or widowed | 84 | 58.35±12.23 |  |  |
| Number of children | 0 | 578 | 57.53±11.55 | 2.419 | 0.089 |
|  | 1 | 531 | 56.20±12.42 |  |  |
|  | ≥2 | 208 | 57.97±11.71 |  |  |
| Mode of employment | Aurhorized personnel | 238 | 58.07±12.07 | 1.289 | 0.276 |
|  | Personnel agency | 338 | 57.23±12.11 |  |  |
|  | Contract worker | 741 | 56.67±11.83 |  |  |

**Table S4 Univariate analyses of work engagement (N = 1317)**

| Variables | | *N* | Mean ± SD | *t*/*F* | *P* |
| --- | --- | --- | --- | --- | --- |
| Age(years) | ≤25 | 472 | 30.48±11.60 | 3.929 | ＜0.001 |
|  | 26-35 | 513 | 32.43±10.26 |  |  |
|  | 36-45 | 234 | 33.69±10.57 |  |  |
|  | ＞45 | 98 | 35.04±10.67 |  |  |
| Gender | Male | 158 | 33.10±11.46 | 1.559 | 0.119 |
|  | Female | 1059 | 31.92±10.78 |  |  |
| Education level | Junior college and below | 492 | 31.27±10.34 | 2.904 | 0.055 |
|  | Bachelor degree | 730 | 32.55±11.06 |  |  |
|  | Master degree or above | 95 | 33.60±12.45 |  |  |
| Professional title | Nurse | 489 | 30.48±10.10 | 10.734 | ＜0.001 |
|  | Nurse practitioner | 518 | 32.62±10.20 |  |  |
|  | Nurse-in-charge or above | 310 | 33.98±12.84 |  |  |
| Departments | Internal medicine | 369 | 31.80±10.92 | 1.463 | 0.188 |
|  | Surgery | 339 | 33.20±10.30 |  |  |
|  | Obstetrics and gynaecology | 136 | 31.63±11.19 |  |  |
|  | Paediatrics | 104 | 33.27±12.91 |  |  |
|  | Emergency | 130 | 31.55±10.47 |  |  |
|  | Intensive care unit | 121 | 30.31±10.91 |  |  |
|  | Others | 118 | 32.36±10.81 |  |  |
| Work experience (years) | 1-5 | 534 | 30.59±11.19 | 7.293 | ＜0.001 |
|  | 6-10 | 476 | 31.55±10.47 |  |  |
|  | 11-15 | 186 | 30.31±10.91 |  |  |
|  | ＞15 | 121 | 32.36±10.81 |  |  |
| Average working hours per week (hours) | ≤40 | 295 | 30.59±11.19 | 7.584 | 0.001 |
|  | 41-50 | 638 | 32.75±10.29 |  |  |
|  | ＞50 | 384 | 33.39±10.40 |  |  |
| Average number of night shifts per month (times) | 0-2 | 141 | 34.74±12.04 | 9.583 | ＜0.001 |
|  | 3-5 | 340 | 33.71±11.51 |  |  |
|  | 6-8 | 555 | 32.41±10.66 |  |  |
|  | ＞8 | 281 | 30.51±10.70 |  |  |

Continued Table S4

| Average monthly income（¥） | ≤3000 | 289 | 29.44±10.43 | 10.413 | ＜0.001 |
| --- | --- | --- | --- | --- | --- |
|  | 3001-6000 | 326 | 31.76±10.49 |  |  |
|  | 6001-9000 | 449 | 32.94±10.63 |  |  |
|  | ＞9000 | 253 | 34.33±11.89 |  |  |
| Marital status | Single | 526 | 31.45±10.39 | 2.029 | 0.132 |
|  | Married | 707 | 32.51±11.09 |  |  |
|  | Divorced or widowed | 84 | 33.42±12.53 |  |  |
| Number of children | 0 | 578 | 31.51±11.69 | 2.224 | 0.109 |
|  | 1 | 531 | 32.89±10.18 |  |  |
|  | ≥2 | 208 | 32.04±10.48 |  |  |
| Mode of employment | Aurhorized personnel | 238 | 33.42±12.38 | 2.484 | 0.084 |
|  | Personnel agency | 338 | 32.35±11.21 |  |  |
|  | Contract worker | 741 | 31.64±10.25 |  |  |

**Table S5 Multiple linear regression analysis of influencing factors of clinical nurses' work engagement (N=1317)**

| Variant | *B* | *SE* | *β* | *t* | *P* | *VIF* |
| --- | --- | --- | --- | --- | --- | --- |
| Constant item | -0.097 | 2.335 | - | -0.041 | 0.967 | - |
| Age | 0.448 | 0.290 | 0.037 | 1.542 | 0.123 | 1.631 |
| Professional title | 0.811 | 0.287 | 0.057 | 2.827 | 0.005 | 1.125 |
| Work experience | 0.028 | 0.280 | 0.002 | 0.099 | 0.921 | 1.649 |
| Average working hours per week | -0.030 | 0.306 | -0.002 | -0.099 | 0.921 | 1.111 |
| Average number of night shifts per month | -0.336 | 0.244 | -0.028 | -1.378 | 0.169 | 1.150 |
| Average monthly income | 0.654 | 0.214 | 0.062 | 3.058 | 0.002 | 1.140 |
| Compassion fatigue | -0.128 | 0.011 | -0.280 | -12.140 | ＜0.001 | 1.474 |
| Professional identity | 0.176 | 0.019 | 0.192 | 9.320 | ＜0.001 | 1.178 |
| Sense of coherence | 0.247 | 0.015 | 0.404 | 16.866 | ＜0.001 | 1.585 |

B, unstandardized coefficient; SE, standard error of the unstandardized coefficient; β, standardized coefficient; t, validity coefficient of the regression; VIF, variance inflation factor; F=162.512, *P*＜0.001, R^2^=0.528.
